# Perception and use of social media by Indonesian adolescents and parents: A qualitative study

**DOI:** 10.3389/fpsyg.2022.985112

**Published:** 2023-01-05

**Authors:** Eka Riyanti Purboningsih, Karlijn Massar, Zahrotur Rusyda Hinduan, Hendriati Agustiani, Robert A. C. Ruiter, Philippe Verduyn

**Affiliations:** ^1^Faculty of Psychology, Universitas Padjadjaran, Bandung, Indonesia; ^2^Faculty of Psychology and Neuroscience, Department of Work and Social Psychology, Maastricht University, Maastricht, Netherlands

**Keywords:** adolescents, parents, social media, Indonesia, qualitative study

## Abstract

Social media are popular among adolescents worldwide, including the global South. The way adolescents use social media is influenced by their own perception of social media but also by how their parents use and perceive social media. This study aims to understand how Indonesian young adolescents (12–15 years old) and parents of adolescents use and perceive social media. For this purpose, we conducted eight focus group discussions and eight semi-structured interviews with 30 Indonesian adolescents and 15 Indonesian parents. Thematic analysis of the qualitative data reveals that both adolescents and parents use social media for social, practical, and pleasure activities. Most adolescents mention that they consider themselves skilled in using social media, while parents consider themselves less skilled. Both adolescents and parents mention that social media offer benefits for adolescents, including emotional, social, and practical benefits. However, adolescents and parents also mention the risks of social media use for adolescents, including social, emotional, and informational risks, as well as the displacement of more meaningful activities. As such, both adolescents and parents do not perceive social media as inherently good or bad but rather as a novel medium that offers benefits for adolescents but also involves several risks to be considered by parents and other relevant stakeholders. This study adds to our understanding of social media use in the global South and offers a theoretical basis for future studies on the impact of adolescents’ social media usage on wellbeing in an Indonesian context. However, future research is necessary to depict possible differences in social media use between Indonesia and other countries in the global South.

## 1. Introduction

Adolescents grow up in a digital world that is markedly different from the world their parents grew up in Palfrey and Gasser (2008). For example, whereas their parents had to go to the library to seek information, adolescents now simply google the information they need on their smartphones. Similarly, whereas their parents had to use a landline or meet in person to connect with friends, adolescents can connect anytime and anywhere using social media applications. Worldwide, more than four billion people use social media and spend more than 2 h daily on these online platforms (Kemp, 2021). Social media refers to platforms that allow users to create and exchange content (Kaplan and Haenlein, 2010), such as Facebook (2.74 billion users), YouTube (2.291 billion users), or WhatsApp (2 billion users) (Kemp, 2021). Social media are popular among all age groups, including adolescents. A study by Rideout et al. (2022) states that adolescents in the United States spend around 1 h 27 min each day on social media. Large-scale studies show that more than 80% of adolescents in the United States and Europe use social media (European Union, 2020; eSafetyCommissioner, 2021; Ofcom, 2021).

Adolescence is a critical period for socio-emotional development (Rosenblum and Lewis, 2008). For example, adolescents have to define their identity, become more autonomous, and learn how to cooperate with others (Margalit, 2010; Newman and Newman, 2015). Social media may help adolescents fulfill these developmental tasks by offering a platform to experiment with different identities (Best et al., 2014; Borca et al., 2015; Valkenburg and Piotrowski, 2017). Moreover, social media may stimulate autonomy (Valkenburg and Piotrowski, 2017) and connectedness (Manago et al., 2020) by offering adolescents a tool to socialize and collaborate with others of their own choosing. However, social media can also negatively influence adolescents (Ansary, 2020). When experimenting with different identities, adolescents may post content they later regret (Allen, 2015; Valkenburg and Piotrowski, 2017), and exposure to carefully selected and edited appearance-related content may lead adolescents to engage in damaging social comparisons and suffer from body dissatisfaction (Frison and Eggermont, 2016a; Sukamto et al., 2019; Vandenbosch et al., 2022). Similarly, exposure to cyberbullying (Handono et al., 2019; Ansary, 2020; Camerini et al., 2020; Asriani et al., 2021; Wiguna et al., 2021), violence (Hogan and Strasburger, 2020), and pornography (Peter and Valkenburg, 2016; Untari et al., 2020) on social media may have negative consequences for adolescents’ wellbeing and development.

Interestingly, adolescents seem to be aware of the positive and negative consequences of social media (OfCom, 2019; Smahel et al., 2020). Qualitative studies on adolescents’ perceptions of social media revealed that they believe that social media allows them to communicate with friends and family (Throuvala et al., 2019a), connect with peers (Davis, 2012), increase social capital (Hayes et al., 2021), and facilitate emotion regulation (Throuvala et al., 2019a). However, adolescents also report that social media sometimes makes them feel anxious, displaces time spend with friends, and creates a social expectation to be always online (Throuvala et al., 2019a).

The consequences of social media use for adolescents depend on how social media are used (Frison and Eggermont, 2016b; Weinstein, 2017; Kross et al., 2020; Verduyn et al., 2022). The way adolescents use social media is influenced by their own perceptions of social media as well as by their social environment. Despite adolescents’ desire for autonomy, they are still strongly influenced by their parents (Collins and Laursen, 2004). Research has shown that parents can impact adolescents’ social media use (Hammer et al., 2021; Stevic and Matthes, 2021) and protect them from possible adverse effects (Shin and Ismail, 2014; Rasmussen et al., 2015; Fardouly et al., 2018).

The degree to which parents affect their adolescent children’s social media use is driven by parental efficacy (Valcke et al., 2010; Jang et al., 2017; Wong and Lee, 2017; Glatz et al., 2018). Parental efficacy pertains to parents’ beliefs about their ability to influence the social media context in which their child grows up (Shumow and Lomax, 2002). The degree to which parents are willing and capable of impacting adolescents’ social media environment depends on their perceptions and skills regarding social media (Hammer et al., 2021). Similar to their adolescent children, qualitative studies revealed that parents perceive both benefits and risks of social media use (Hayes et al., 2021).

In sum, several (qualitative) studies have assessed how adolescents and their parents perceive social media. However, these studies have typically been conducted in the global North, and it is unclear to what degree these findings generalize to countries in the global South (Magis-Weinberg et al., 2021; Ghai et al., 2022), primarily because of how a person uses social media depends on subjective and cultural contexts (Parry et al., 2022). Social media are popular worldwide, including in Southeast Asia (United Nations International Children’s Emergency Fund [UNICEF], 2020b; Tankovska, 2021b). Indonesia is the most densely populated Southeast Asian country, and both the social media penetration level (61.8%) and the amount of time spent on social media (>3 h/day) are above the worldwide average (Tankovska, 2021a). In Indonesia, 93.52% of adolescents use social media (Kementerian Komunikasi dan Informatika Republik Indonesia [KOMINFO], 2017). In terms of culture, Indonesia is often used as an example of a collectivist culture (Yamaguchi et al., 1995; Sugimoto, 1998) which is different from most countries in the global North. In a collectivist culture, respect for hierarchical social structure is important (Riany et al., 2017), and the use of social norms that emphasize duty, obedience, conformity, and interdependence in society is widespread (Triandis et al., 1990). These beliefs may affect Indonesians’ motivations for using social media. However, it should be noted that due to the influence of globalization, individualistic cultural orientations begin to emerge in Indonesian adolescents (Muttaqin, 2020).

Despite the massive adoption of social media in Indonesia, qualitative studies on adolescents’ perceptions of social media are rare. Several exceptions have to be noted, but these studies are limited to specific social media platforms (e.g., Facebook or Instagram) (Kristanto, 2010), particular content (e.g., pranks or hoaxes) (Astuti and Mustofa, 2020; Barito, 2021; Moulita and Lubis, 2021), or contexts (e.g., education) (Nurdiansyah and Lestari, 2021). Similarly, qualitative studies on Indonesian parents are limited to particular social media platforms (e.g., TikTok or Instagram) (Juwita, 2019; Sari and Basit, 2020).

Therefore, the present study aims to understand how Indonesian young adolescents (12–15 years old) and parents of adolescents think about and use social media. For this purpose, we conducted focus group discussions and semi-structured interviews. We asked adolescents (parents) to report on their own perceptions and use of social media and those of their parents (adolescent children). The present study complements prior research on social media perceptions that have been predominantly conducted outside an Indonesian context in the global North. Moreover, the present study provides a framework that can be used for future studies on social media use in Indonesia. Indeed, this study describes Indonesian adolescents’ social media use and engagement. Social media motivation and engagement are of major importance when explaining the relationship between social media use and adolescents’ wellbeing (Valkenburg, 2022). Furthermore, it is vital to understand how adolescents and parents perceive social media are taking cultural context into account. These perceptions impact how adolescents and parents use social media, how parents regulate their adolescent children’s social media use, and adolescents’ responses to these regulation strategies^1^.

## 2. Materials and methods

### 2.1. Study setting

The location of this study was the city of Bandung, West Java, Indonesia. The large majority (73.7%) of Indonesians use the internet, and 56.4% of all users are on Java Island. Moreover, of the six provinces of Java, the internet is mainly used in West Java (17.9%) (Asosiasi Penyelenggara Jasa Internet Indonesia [APJII], 2020). Bandung is the capital of West Java and has a higher internet penetration rate (74.1%) than rural areas (61.6%) in West Java (Asosiasi Penyelenggara Jasa Internet Indonesia [APJII], 2018).

### 2.2. Participants

*Adolescents*. Adolescents were allowed to participate in the study if they were 12–15 years old and had used social media in their daily lives during the past year. We focused on this specific age group because it constitutes the first phase of adolescence, and during this period, several major biological, cognitive, and socio-emotional changes occur. Since the rise of social media, these major developmental changes now take place in an increasingly digital environment. It is therefore of important to examine the interaction between developmental changes and social media in early adolescents.

**FIGURE 1:**
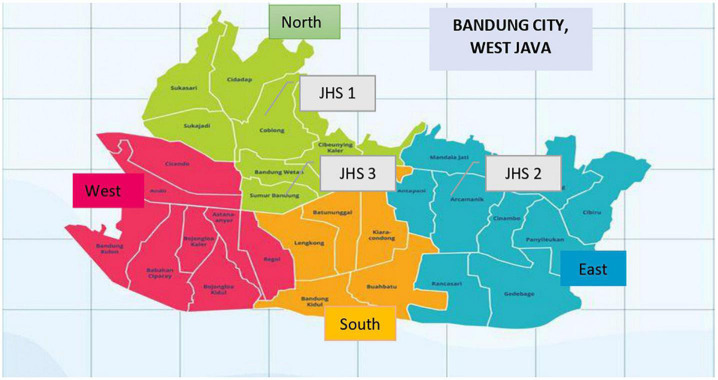
The school location map (https://www.shutterstock.com/en/image-vector/illustration-vector-map-bandung-jawa-barat-1854052687).

We approached four public junior high schools in Bandung randomly to recruit adolescents, and three schools agreed to participate. Since the education system in Indonesia uses a zoning system (i.e., students from a particular junior high school come from the surrounding neighborhood), the characteristics of students of each three participating junior high schools are different (see Figure 1). Each school selected and invited ten adolescent students to participate. All selected participants (*N* = 30) were willing to participate in our study and provided consent. Upon collecting data from these thirty participants, data saturation was reached. Half of the adolescent participants were male. Participants were on average 13.7 years old. Participants received an honorarium for a total value of two dollars in return for their participation.

*Parents*. Parents were allowed to participate if they had adolescent children (12–15 years), lived with their children, and had used social media themselves during the past year. We used two procedures to recruit parents. First, we organized an educational seminar on digital parenting and advertised this event through parent-teacher associations by distributing an invitation letter. Thirty-five parents participated in the event, but only seven of them had adolescent children. We invited those seven parents to participate in our study (which took place before the educational seminar), and all of them provided their consent. Parents received an honorarium for a total value of four dollars in return for their participation in this study. Second, we recruited participants by posting the study information on social media. Eleven parents who did not have a close friendship with the researchers expressed their interest, but three parents eventually did not participate. Using these two approaches, we recruited a total of 15 parents. All parents had multiple children and were asked to report on their oldest adolescent child. Upon collecting data from 15 parents, data saturation was reached and no additional parents were recruited. The sample of parent participants consisted of 14 mothers and one father and they were on average 38.6 years old. Their occupation was a housewife (53.3%), government employee (33.3%), or private employee (13.3%).

### 2.3. Procedure

The study protocol was approved by the ethical review committee from Universitas Padjadjaran and data were gathered from November 2019 to August 2020. To collect data, we used focus group discussions (FGD) with adolescents and parents and semi-structured individual interviews (only parents). All interviews were conducted in Bahasa Indonesia, audio-recorded after obtaining participants’ consent, and transcribed verbatim by the facilitator.

*Focus Group Discussions*. Eight focus group facilitators were trained by one of the researchers on how to lead the sessions. The facilitators were psychology students who had completed the Interview and Focus Group Discussion courses. One of the researchers explained the interview protocol and provided prompts (i.e., a series of questions to follow up on the central questions). This researcher was also at the FGD’s location to supervise the data collection. During the FGDs, the facilitator posed a series of questions and only interrupted participants to (a) ensure that the conversation did not go off-topic, (b) involve less active participants, or (c) ask for clarification when participants’ answers were unclear. We conducted six focus group discussions with five adolescent participants in each group (the teacher was not present in the room), one focus group discussion with three parents, and one with four parents. The FGDs were conducted in a class (adolescents) or seminar room (parents). The FGDs with the adolescents lasted 35–60 min and those of the parents 40–60 min.

*Individual interviews*. Due to the outbreak of COVID-19, the focus group interviews with parent participants could no longer take place, and we collected additional data from 8 parents by conducting individual interviews over the telephone. One of the researchers conducted the interviews for 25–30 min.

### 2.4. Materials

*Adolescent Participants*. Before the focus group discussions took place, adolescent participants provided socio-demographic information by completing an open-ended brief paper and pencil questionnaire assessing (a) their age, (b) their parents’ occupation, (c) the electronic devices they use to communicate, (d) the devices their parents use to communicate, (e) their activities on social media, and (f) their parents’ activities on social media.

During the focus group discussions, adolescents were asked six questions: (a) What is your most significant reason for using social media? (b) What are the consequences (both positive and negative) you experience when using social media? (c) To what extent are you able to use social media? (d) What activities do your parents engage in on social media? (e) To what extent are your parents able to use social media? (f) To what extent could social media contribute to your happiness? Regarding the last question, it is notable that after participants provided a general evaluation, they were asked to provide reasons why they believe social media contributed to their happiness or not.

*Parent Participants*. Similarly to adolescent participants, parents first completed an open-ended brief paper and pencil questionnaire assessing (a) their age, (b) their occupation, (c) the number and age of their children, (d) the electronic devices they use to communicate, (e) the devices their children use to communicate, (f) their activities on social media, and (g) the activities of their children on social media. When interviews rather than focus group discussions took place, the interviewer read the questions to the participants and participants provided verbal responses.

During the focus group discussions and individual interviews, parents were asked five questions: (a) What activities do you engage in on smartphones, the internet, and social media? (b) To what extent are you able to use a smartphone, the internet, and social media? (c) What activities do your adolescent children engage in on smartphones, the internet, and social media? (d) What are the consequences (both positive and negative) for your adolescent children of using smartphones, the internet, and social media? and (e) To what extent do your adolescent children have the ability to use smartphones, the internet, and social media?

We asked questions starting from smartphones to the internet to social media to make it easier for parents to distinguish between them (i.e., between activities carried out on smartphones, the internet, or social media). However, during data collection, we noticed that some parents did not distinguish between social media use vs. internet or smartphone use. For example, when asked about the activities they engage in on social media, parents sometimes described activities they do on their smartphone (like listening to music using a music app) or on the internet (like googling) that are not considered social media use. In our data analysis and results section, we follow parents’ conceptualization of social media rather than excluding responses related to general smartphone or internet use. However, most parents’ responses to the social media questions were consistent with traditional definitions of social media use.

### 2.5. Data analysis

**FIGURE 2:**
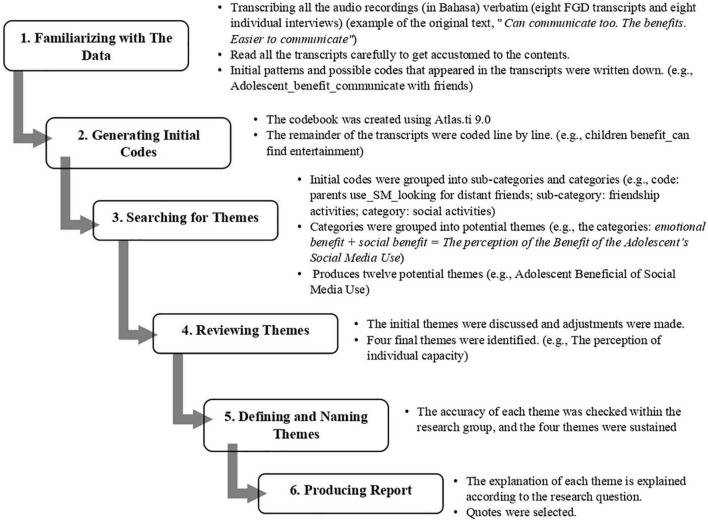
Six-step of thematic analysis (Braun and Clarke, 2006) (https://www.shutterstock.com/en/image-vector/illustration-vector-map-bandung-jawa-barat-1854052687).

Data analysis was done using the 6 phases of thematic analysis (Braun and Clarke, 2006). Figure 2 describes the process that was conducted. The first phase (familiarizing with the data) started with transcribing all the audio recordings (in Bahasa) verbatim (eight FGD transcripts and eight individual interviews). The transcripts were saved in MS Word. Before data analysis was started, the first author read all the transcripts carefully to get accustomed to the contents. If some words or sentences were unclearly written (usually because the recording was less audible), the researcher listened to the recording to clarify the text. During this phase, initial patterns and possible codes that appeared in the transcripts were written down.

In the second phase—generating initial codes—the codebook was created using Atlas.ti 9.0. An inductive approach to coding was used. To verify the validity of the initial codebook, the inter-coder agreement was established. The first author and an independent coder, who was not involved in the data collection phase, coded two transcripts (one parent and one adolescent transcript) separately and discussed their codebooks afterward. Disagreements were resolved by first re-reading the section of the text and then discussing the best coding option. After the intercoder agreement was established, the codebook was finalized, and the remaining transcripts were coded by the first author.

Subsequently, in phase 3 (searching for themes), initial codes were grouped into sub-categories and categories. For example, the initial codes *adolescent_benefit_Online Game_children looking forward to play Online Game with friends* and *adolescent benefit_SM_well connected with friends* were grouped under the category *companion benefits*. This process resulted in sixteen categories. Categories were grouped into potential themes by looking at their mutual relationships. Next, in Phase 4 (reviewing themes), the research team members discussed these categories, making adjustments until a consensus was reached about the final set of themes. In this way, four final themes were identified and these themes were divided into more specific sub-themes. Codes that did not fit within these categories or themes and were deemed irrelevant for a new theme were discarded from the analysis. In phase 5 (defining and naming themes), the accuracy of each theme was checked, and the four themes were sustained. Eventually, in phase 6 (producing the report), the meaning of each theme was explained according to the research questions. Quotes were included to strengthen and illustrate our interpretation. A visualization showing the codes and how those codes building related themes is described in Table 1.

**TABLE 1:** Summary of the coding process.

Theme	Subthemes	Categories	Sub-categories
**Theme 1: Adolescents’ and parents’ social media activities**	a) The adolescent’s social media activities: Adolescent’s perspective b) The adolescent’s social media activities: Parent’s perspective	**Social activities**	**a) Communication activities** (Adolescent) “*It’s the same as being able to call my parents. So, we can contact each other. We are not living in the same house, so that we can call each other*” (Ad. Male23_15 yo) (Parents) “*Like chatting*…*asking something to her friends*” (Mother04_29 yo) **b) Friendship activities** (Adolescent) *“Yes, for add friends, for example, I’m from Manglayang, then she/he is from…”* (Ad. Female18_14 yo)
		**Practical activities**	**a) Commerce activities** (Adolescent) *“For online shopping”* (Ad. Female19_15 yo) (Parent) “S*ometimes he likes to buy something online, anything he wants*” (Mother03, 37 yo) **b) Information-seeking activities** (Adolescent) “… *looking for information, for example, about soccer”* (Ad. Female28_14 yo) (Parent) “*Everything about K-Pop until she knows the details”* (Father14_45 yo) **c) School and study activities** (Adolescent) *“For example, if there is an art festival at school, then there must be tasks to be done, right. Well, you have to share the tasks”* (Ad. Female27_14 yo) (Parent) *“Usually he looks at class groups, for example, what assignments or announcements are there”* (Mother07_36 yo)
		**Pleasure activities**	**a) Entertainment activities** (Adolescent) *“Well, if I am not in the mood, I like to open accounts like awreceh”* (Ad. Female02_13 yo) (Parent) *“She likes watching YouTube since she is in elementary school. She likes to watch dance video”* (Mother04_29 yo) **b) Online gaming activities** (Adolescent) “… *usually I play a game”* (Ad. Male11_14 yo) (Parent) *“He plays online games*” (Mother01_38 yo)
	c) The parent’s social media activities: Adolescent’s perspective d) The parent’s social media activities: Parent’s perspective	**Social activities**	**a) Communication activities** (Adolescent) *“share news”* (Ad. Male30, 14 yo) (Parent) *“for communication”* (Mother10, 39 yo) **b) Friendship activities** (Parent) *“We can look for distant friends on Facebook, right?”* (Mother04, 29 yo)
		**Practical activities**	**a) Work activities** (Adolescent) *“For work transaction”* (Ad. Male23_15 yo) (Parent) *“At that time, I wanted to have an IG live so that we could talk about a certain topic*… *then let’s just make an IG”* (Mother12, 40 yo) **b) Information-seeking activities** (Adolescent) “W*atching update of news on YouTube, like news of today*” (Ad. Male11_14 yo) (Parent) *“We search for information so that our children don’t fall into bad things, right?”* (Mother01, 38 yo) **c) Commerce activities** b) (Parent) *for ordering in “Gojek or Grab”* (Mother05, 41 yo)
		**Pleasure activities**	**a) Entertainment activities** (Adolescent) *“She watches Korean drama all the time”* (Ad. Female04_13 yo) (Parent) “*So, there are several Instagram accounts that I follow. Usually related to hobbies”* (Father14, 45 yo)
**Theme 2: The perception of individual capacity**	a) The perception of adolescent’s capacity: Adolescent’s perspective b) The perception of adolescent’s capacity: Parent’s perspective	**Children’s capability use SM**	**a) Operational_Capability** (Adolescent) “*Make or edit the video so it can be more interesting*…” (Ad. Female02_13 yo) (Parent) “*From studying (the smartphone), my child becomes a teacher on his own so that he can do it by himself*” (Mother05, 41 yo) **b) Content_Evaluation_Capability** (Adolescent) “*able to tell the difference between good and bad*” (Ad. Male12_14 yo) (Parent) *He already knows that too. His friends are playing GTA too, so he chose not to because he knows it’s too rough*” (Mother08, 40 yo) **c) Self-control_Capability** (Parent) *“So, in the end, he was given a smartphone to do that. well, for himself, he could manage it himself*” (Mother08, 40 yo)
		**Children’s incapability use SM**	**a) Operational_Incapability** (Adolescent) “*Still don’t understand the function of each of the icons*” (Ad. Male24_15 yo) (Parent) “*There’s a new feature and she doesn’t know. Then, she asked me too*” (Mother10_39 yo) **b) Self-Control_Incapability** (Adolescent) “… *but sometimes (when I use social media), I still forget about the time*” (Ad. Female08_13 yo) (Parent) “*My homework is more about controlling him. If he’s invited by his friends (to play an online game), he’s delighted. Sometimes I have to remind him, “Have you finished your homework?”* (Mother12, 40 yo)
	c) The perception of parent’s capacity: Adolescent’s perspective d) The perception of parent’s capacity: Parent’s perspective	**Parents’ capability use SM**	**a) Operational_Capability** (Adolescent) “*Yes, my father is usually the one who often uses it (social media)*” (Ad. Male29_15 yo) (Parent) “*because I can still solve it if I have trouble, I can fix it myself. Sometimes, my husband is more clueless*” (Mother15, 40 yo) **b) Content_Evaluation_Capability** (Adolescent) “*Because he must already know the negative and the positive. He is already an adult*” (Ad. Male26_15 yo) **c) Time-management_Capability** (Adolescent) “*For example, if she has free time, she plays the game but only for a while. Play then go back straight to work again*” (Ad. Female08_13 yo)
		**Parents’ incapability use SM**	**a) Operational_Capability** (Adolescent) “*They still ask a lot of questions*” (Ad. Male16_14 yo) (Parent) “*Well, I only use Whatsapp and video call only. It is just for business, calling my husband, communicating with others*” (Mother03, 37 yo)
**Theme 3: Perceived benefits of social media use for adolescents**	a) Adolescent’s perspective b) Parents’ perspective	**Emotional benefits**	**a) Mood Enhancement_Benefit** (Adolescent) “*Because on social media, there are videos on IG that can lift the mood. Friends keep on chatting also making the mood go up*” (Ad. Female04_13 yo) (Parent) “*She may share maybe her emotions like sometimes telling stories with her friends.*” (Mother13, 44 yo) **b) Motivational_Benefit** (Adolescent) “*Yes, YouTube as a source for inspiration. I like automotive, so I was inspired to build a repair shop*” (Ad. Female18_14 yo) **c) Entertainment_Benefit** (Adolescent) “*Keep on filling your spare time*” (Ad. Male12_14 yo) (Parent) “*Indeed, the smartphone was usually used to play a game. It’s just for entertainment, that’s okay.”* (Mother01, 38 yo)
		**Social benefits**	**a) Companion_Benefit** (Adolescent) “*So*… *no, it’s not that lonely when you don’t have friends*” (Ad. Male16_14 yo) (Parent) “*When he plays (online game) together, it’s time to socialize. Because that’s where he’s talking to his friends, right*?” (Mother09, 39 yo) **b) Relational_Benefit** (Adolescent) “*We can cooperate with others*” (Ad. Female22_14 yo) **c) Communication_Benefit** d) (Adolescent) “Y*ou can give news to distant family via WhatsApp. I think it is easier that way*” (Ad. Female09_12 yo)
		**Practical benefits**	**a) Enhance_Knowledge_Benefit** (Adolescent) “*We are more insightful, so we can find information that we haven’t got yet*” (Ad. Male25_14 yo) (Parent) “*My son likes bicycles, so he fiddled with his bike with clay from YouTube, and likes to make toys out of cans and plastic bottles from YouTube*” (Mother06, 36 yo) **b) Educational_Benefit** (Adolescent) “*Yes, it’s like. for example. for school lessons, seeking knowledge, from YouTube*” (Ad. Female13_14 yo) (Parent) “*But sometimes I’ve just seen it through the internet how to find it (an answer to her children’s homework)”* (Mother01, 38 yo) **c) Facility_Benefit** (Adolescent) “*Sometimes it gets easier to do many things*” (Ad. Female07_13 yo)
**Theme 4: Perceived risks of social media use for adolescence**	a) Adolescent’s perspective b) Parents’ perspective	**Displacement risks**	**a) Activities_Disturbance_ Risk** (Adolescent) “*You see*… *like I start forgetting the world*” (Ad. Female10_13 yo) (Parent) *Not focus when studying* (Mother11, 39 yo) **b) Dependence_ Risk** (Adolescent) “*Become addicted*” (Ad. Male16_14 yo) (Parent) “*So, he still needs help on that part (managing time when playing online game)”* (Mother08, 40 yo) **c) Financial_ Risk** (Adolescent) “W*asting our quota*” (Ad. Female09_12 yo) (Parent) “*But the negative is wasting the quota. Because I am the one who bought it*” (Mother03, 37 yo)
		**Social and emotional risks**	**a) Relational_Risk** (Adolescent) “*So more often in my room playing (online game). When there is a family gathering, I prefer in the room*” (Ad. Female03_13 yo) (Parent) “*As time goes on with new technology, this increases children’s insensitivity to the environment*” (Mother11, 39 yo) **b) Friendship_ Risk** (Adolescent) “*Yes, if you have a problem with friends. They will make a snide remark*” (Ad. Female02_13 yo) **c) Cyberbullying_ Risk** (Adolescent) *receiving negative comments on social media, for example, when you were going out with your parents. Often, we take pictures with mom and dad. They said, “Uuugh, you pretend to be a happy family”* (Ad. Female07_13 yo) **d) Discouragement_ Risk** d) (Adolescent) “*But I’m also happier when I do my hobby anyway. It’s just a hobby, instead of playing with gadget*” (Ad.Male05_ 12 yo)
		**Informational risks**	**a) Negative exposure _ Risk** (Adolescent) “*It pushes us to do sinful things. So, if we open the internet, we are going to open sites that are. not that good*” (Ad. Male12_14 yo) (Parent) “*Sometimes there is manga like that, but there are pornographic elements*” (Mother13, 44 yo) **b) Privacy_ Risk** (Adolescent) “*The downside is that other people can access our personal information*” (Ad. Male30_14 yo)

When presenting the results, we focus on describing the adolescents’ point of view, and the parents’ point of view will be conveyed to the extent that it is different from the adolescent’s point of view.

## 3. Results

### 3.1. Theme 1: Adolescents’ and parents’ social media activities

The most popular social media applications among adolescents were Instagram (93.3%), followed by WhatsApp (86.7%), YouTube (63.3%), Line (60%), and Facebook (23.3%). All parents reported using WhatsApp (100%), followed by Facebook (80%), Instagram (53%), and YouTube (20%). While adolescents and parents made use of partially different platforms to engage with social media, the activities they conducted show a large overlap. Specifically, adolescents and parents mentioned using social media for (1) social activities, (2) practical activities, and (3) pleasure activities.

*Social activities.* Both adolescents and parents mentioned using social media for social activities. This includes communicating with others but also maintaining, rekindling, or seeking friendships. For example, adolescents and parents use social media to update stories, post on Instagram or Facebook or read other people’s posts. When asked whom they communicated with, adolescents’ responses indicated they communicate mainly with friends and parents. The content of the conversations was broad, ranging from gossiping with friends to make up when they had argued with their parents. Parents added that adolescents also communicate with teachers about school activities. Parents mainly communicate to get information from their children’s school, family, relatives, and friends.

*Most of all, I am communicating with my friends, ask them what they are doing. Also, with my extracurricular friends* (Ad.Male12, 14yo).

*We exchange news. On Facebook, I look for friends I have not seen in a long time* (Mother04, 29yo).

*Practical activities.* Adolescents and parents often use social media for practical purposes. Practical activities include commerce activities (e.g., buying or selling goods), information-seeking activities, and school or work activities. Adolescents seek information in line with their interests, such as their hobbies or favorite music groups. Parents search for information on people (e.g., family, relatives, or friends), children’s school activities, national or international news, religious topics, and recipes. Adolescents also use social media to support their school activities (e.g., asking questions about homework or discussing school subjects), while parents use social media for work (e.g., use Instagram Live to discuss topics related to their work).

*Mostly, I watch the channel on YouTube about repairing or modifying motorbikes or motorbike machines. I want to be a motorcycle racer* (Ad. Male17, 13 yo).

*Then at one moment, we wanted to have an* Instagram *live discussion. We would talk about a particular topic on* Instagram *live. I didn’t have an Instagram account at that time. So, I made it to support my work* (Mother12, 40 yo).

*Pleasure activities.* Both adolescents and parents use social media to seek entertainment (e.g., watching YouTube). Moreover, social media is used by adolescents and parents to play games. The nature of the games differed somewhat. While adolescents mainly play Massively Multiplayer Online Roleplaying Games (MMORPG) with their friends, parents play life-simulation games or offline mobile games.

*I like to watch Youtube, looking at videos like ASMR (autonomous sensory meridian response). You know, how people make a sound while they eat. I also like to watch pranks video and music* (Ad.Female13, 13 yo).

*So, there are several Instagram accounts that I follow. Usually, it related to hobbies* (Father14, 45 yo).

### 3.2. Theme 2: The perception of individual capacity

This theme describes how parents and adolescents perceive their abilities to use social media. Although the activities carried out by adolescents and parents on social media are largely the same (see theme 1), their perceptions of their ability to use social media are quite different.

Most adolescents report feeling skilled in utilizing the various options of social media applications, including voice calls, chatting, commenting, posting, and editing photos or videos. They reported learning to use these features by themselves. By often using social media, they gradually got more familiar with these applications. Only a few adolescents stated that they did not use certain features (e.g., using Instastory, live video, or filters) often since they did not use social media that often. One limitation adolescents mentioned is that most applications utilize the English language and that their English language skills were insufficient to use social media optimally. Some adolescents felt not only skilled to use social media (operational capability) but also said they felt capable of evaluating the content of social media (content evaluation capability):

*I am already able to tell the difference between good and bad on social media* (Ad. Male01_ 13 yo).

Overall, adolescents consider themselves capable of using social media, and parents agree. However, most parents mention that their adolescent children lack self-control, especially in time management, when they play online games. Parents still need to remind adolescents to stop or limit their social media time. Adolescents seem less aware of their lack of time management skills since only a few of them mentioned this issue:

*Because if I forget about time, my parents will always scold at me. I rarely did my homework during a specific period, and a bad thing happened. I was not in the top three of my class twice* (Ad. Female10, 13 yo).

Meanwhile, most parents have a low opinion about their own social media skills, and adolescents confirm this. Many parents state that they still have to ask their adolescent children a lot about how certain features of social media function. Parents said that they do not have much time to learn new features on social media due to being busy taking care of home chores and feel that their needs are already covered by the basic social media features they use on a daily basis.

*If I didn’t understand, I asked my child. For example, if we were searching in Google and there was something I did not understand, I would ask him. Then he said, “uh, mama, it should be like this. You are illiterate in technology”* (Mother06, 36 yo).

*The important thing is that you can call or chat via WhatsApp. That is the important thing for me. I really don’t have much free time, so I can’t learn all the features* (Mother05, 41 yo).

### 3.3. Theme 3: Perceived benefits of social media use for adolescents

The third theme describes the benefits of social media use for adolescents according to adolescents and parents. Three classes of benefits can be distinguished: emotional, social, and practical benefits.

*Emotional benefits*. The adolescents mentioned that social media could enhance their mood as it can provide them consolation and happiness when playing online games, watching YouTube (music videos, sports, and pranks), viewing funny accounts on Instagram, or using TikTok. They said social media relieved their boredom and are always there to fill their free time. Additionally, getting comments or likes on their posts on social media, getting funny video posts from friends, or just chatting with their friends provides happiness and positive emotions, but also provides an outlet to simply express emotions:

*Social media is like*…*isn’t it just an outlet (emotionally)? Heehee (Laughing)* (Ad. Female18, 14 yo).

*I like playing social media because it doesn’t make me feel bored, especially when I have nothing to do* (Ad.Female04, 13yo).

*Social benefits*. Most adolescents mention that social media make it easier to contact and be contacted by others. This allows them to feel close and always feel connected with their friends and family, even if they are in faraway places. Social media help them exchange news, information, or gossip about friends (or family members) and events at school, which can strengthen their existing relationships. Even more, social media assist them in broadening their social network by having many online friends and working together with others. Having online friends on social media and offline friends helps adolescents get rid of feelings of loneliness. Parents also confirm these benefits of social media for their adolescent children. Specifically, they add that playing online games supports adolescents’ social development (e.g., maintaining and expanding relationships and learning social roles).

… *Together with his friends, but the opponent is someone else. We do not know the opponent, but he plays with his friends. So, when he plays together, it is socialization time. Okay, I let him play every day, like for two hours. That is (when playing online games) where he chats with his friends, “Come here, let us make a strategy.”* (Mother09, 39 yo).

*Then the second one, of course, feels like he has a friend. He feels like he can laugh with his friends while playing (online games). Joke with each other* (Mother12, 40 yo).

*Practical benefits*. Both adolescents and parents state that social media (mainly YouTube) help adolescents gain practical knowledge (hobbies, learning a new language, get to know about trending issues) and assist adolescents in understanding school lessons. By knowing this information, adolescents feel they can be more careful so that bad events reported on social media do not happen to them. Parents add that social media help adolescents’ psychological development, including their self-confidence (e.g., being a leader when playing an online game with friends), autonomy (e.g., searching for information independently), creativity (e.g., being able to fiddle with their bikes from watching YouTube), and problem-solving (e.g., develop online game strategies with friends).

*On social media, there is news, for example, the news that informs someone something bad has happened. So, we know how to act so that the negative things don’t happen to us* (Ad. Female22, 14 yo).

*Once I asked my son the reason he played online games. My son said, “I’m the one my friends could depend on. If I weren’t there (playing the online game together), they would have lost.” He looks happy if he can help his friends (to win the game) by being the online game leader. It makes him confident. He also learns to develop a strategy to win the game. So, when he uses his plan and then they win, the joy increases* (Mother09, 39 yo).

### 3.4. Theme 4: Perceived risks of social media use for adolescence

Social media use offers benefits to adolescents. However, both adolescents and parents also recognize the risks of social media use for adolescents.

*Displacement Risks*. Adolescents most often mention the risk that social media may disrupt their daily activities and learning process. Specifically, they forget about time, forget to eat, and procrastinate when asked to do something by their parents. They sometimes watch useless videos on YouTube, which prevents them from studying or makes them do their school assignments carelessly. Social media could also be addictive. It makes adolescents want to use social media more and more. Parents also confirm that adolescents cannot control themselves and spend too much time using social media or playing online games. Due to this excessive use, some adolescents also suffer health-related drawbacks, such as dizziness, eyesight damage, radiation from the smartphone, and sleep disturbance (lack of sleep and oversleeping). Some adolescents also report that they spend too much money due to social media, either spending internet quota or purchasing goods advertised on social media.

*Out of quota. Then you’ll get addicted to it. You want to fill up (the quota), keep filling, keep filling. Spending (quota) away is bad, but stopping (from using) it is challenging* (Ad. Female18, 14 yo).

*Social and Emotional Risks*. While having friends on social media is considered a benefit, these same friends can also harm adolescents. Friends on social media can cause adolescents to be upset or in a bad mood when they do things adolescents do not like. Several adolescents also get cyberbullied on social media. However, parents did not mention cyberbullying as a social media risk for adolescents.

*You receive a nasty comment. For example, when we post a photo, then you will get a nasty comment* (Ad. Male06, 14 yo).

Some adolescents complain that making and maintaining friendships on social media reduces face-to-face communication between them and the people around them. They prefer to be alone or in their room and do not participate in activities with family. Parents also agree with this, and they add that technology makes adolescents less sensitive to their surroundings.

*It’s more to time. It’s impactful (on time). So, you spend more time in your room, playing (online games). If there is a family gathering (in your house), you will prefer to stay in your room* (Ad. Female03, 13 yo).

*Yes, it’s just that if he plays with his gadgets, it will reduce his socialization* (Mother11, 39 yo).

Adolescents said that sometimes social media make them bored. Nothing catches their attention on social media, so they prefer to play with their friends.

*ehm, sometimes social media is*… *sometimes. I am entertained but not very engaged, sometimes making me feel bored. I prefer to be outside, playing outside with friends. We are not playing with smartphones. So, it’s like playing Uno. It is like so much fun, that’s it. We don’t play with our smartphones* (Ad. Female13, 14 yo).

*Informational Risks*. Adolescents and parents agreed that social media could lead to negative content exposure for adolescents, either intentionally or unintentionally. Some adolescents may surf inappropriate websites to look for prohibited information or accidentally receive negative information, such as pornography or online dating, when they are searching for information related to school lessons. Some adolescents are also worried about the privacy of their personal information.

*I was searching at that time, suddenly someone sent me info about online dating, or while I was searching for information I needed, suddenly images or links to adult sites appeared* (Ad. Female18, 14 yo).

## 4. Discussion

Social media has become a critical developmental context for adolescents (Cohen et al., 2021). Although much is already known about adolescents’ use and perception of social media (Boer et al., 2021; Boniel-Nissim et al., 2021; Course-Choi and Hammond, 2021), there is a dearth of research about this topic in an Indonesian context. Therefore, the aim of the present study was to understand how Indonesian adolescents and parents use social media. Moreover, we examined how adolescents and parents perceive the consequences of social media use for adolescents.

### 4.1. How do Indonesian adolescents and parents use social media?

The activities adolescents and parents engage in on social media are quite similar. By posting messages or stories on Instagram or Facebook, sending texts on WhatsApp or reading other people’s posts, adolescents but also parents communicate with others, seek friendships and maintain relationships. Adolescents mainly communicate with friends, parents and teachers, while parents mainly communicate with family, relatives, friends, and their children’s teachers. Social media do not only support adolescents and parents for social activities but also for practical activities, such as buying or selling goods, seeking information related to hobbies, and participating in school or work activities (e.g., asking questions about homework or doing business). Moreover, both adolescents and parents use social media to seek entertainment by, for example, watching YouTube or playing games.

The present findings on adolescents’ and parents’ social media use in an Indonesian context are largely compatible with prior studies on social media use. In prior research on social media use by adolescents across the globe, adolescents were typically found to use social media for communication, entertainment and education (OfCom, 2019; Smahel et al., 2020; United Nations International Children’s Emergency Fund [UNICEF], 2020b). Moreover, in a study conducted by GlobalWebIndex (2019), generation X (37–55 years old) were found to use social media to stay up-to-date on news and events, keep in touch with friends, fill up their spare time, and network with others. Furthermore, adults who were also parents were found to use social media to communicate with family members and connect with the community (Doty and Dworkin, 2014). Even though the present results indicate that social media are similarly used in Indonesia as compared to other countries, it is notable that we found only in Indonesia, adolescents use social media to generate an income. The study conducted by United Nations International Children’s Emergency Fund [UNICEF] (2020b) supports this result. Indonesian adolescents are for example, buying and reselling clothes online, as well as making trinkets and selling them through apps such as Instagram and Facebook.

The present findings on Indonesian adolescents’ and parents’ social media use are compatible with the Uses and Gratification Theory (Katz, 1974). According to this theory, people have their own motivations when choosing and using social media, including staying in touch with friends (Park et al., 2009) and family members (Chen, 2011), seeking for information (Grasmuck et al., 2008; Bulut and Doğan, 2017), or looking for entertainment (Barker, 2009; Bulut and Doğan, 2017). If users believe that selected social media platforms fulfill these motivations and they experience benefits from social media use, they will continue using the social media platform(s). For adolescents, social media may help fulfill needs that are critical for the fulfillment of their development tasks. Adolescents can manage their personal information, create an online identity to achieve peer acceptance (Dunne et al., 2010; Magis-Weinberg et al., 2021), communicate with friends to maintain friendships, and, at the same time, develop autonomy (Borca et al., 2015).

### 4.2. How do Indonesian adolescents and parents perceive their social media skills?

The adolescents consider themselves capable of using social media, and parents agree with this assessment. Adolescents feel capable of utilizing various options of social media applications, including voice calls, chatting, commenting, posting, and editing photos or videos. A prior study conducted by Kusyanti et al. (2018) on Indonesian adolescents supports the results of the present study. They found that Indonesian adolescents believe that social media is very easy to use and that it does not require much effort to obtain benefits from social media use. Similar findings were also obtained in research in the global North. In a study conducted by EU Kids Online in 19 countries (Smahel et al., 2020), it was found that the majority of the children have a high opinion of their operational (e.g., saving photos, changing privacy settings) and social skills (e.g., sharing information) on social media.

It is notable that in our study Indonesian adolescents talked more often about skills to foster social relations (e.g., chat, calls, commenting) than skills to engage in self-expression (e.g., posting and editing photos or videos). Social media allows for expressing one’s identity (Andalibi et al., 2017; Rambaree and Knez, 2017). However, for Indonesian adolescents who have an interdependent self-construal, social connectedness maybe even more important as it allows them to maintain harmonious relationships and feelings of mutual dependence with others (Muttaqin, 2020).

The parents’ ability to use social media contrasts with the ability of adolescents. Parents and adolescents agree that adolescents are more capable of using social media than parents. This phenomenon has been demonstrated before mainly in low and middle-income countries, where parents lag behind in adopting and using technology (Livingstone and Byrne, 2018). To help out, children often teach their parents how to use computers, mobile phones, and the Internet (Correa, 2014; Magis-Weinberg et al., 2021). Parents’ lack of social media skills may have negative consequences as parents significantly influence adolescents’ lives (Collins and Laursen, 2004), especially in an Indonesian context where adolescents often conform to parental rules and hopes as an embodiment of respect (Muttaqin, 2020). Parents can positively impact adolescents’ social media use (Hammer et al., 2021; Stevic and Matthes, 2021) but this is less likely in the absence of parental social media skills as skills toward social media underlie the willingness and capability to manage adolescents’ social media environment (Hammer et al., 2021). Parents who lack social media skill may even excessively restrict their children’s social media use so that their children cannot develop the necessary skills and technical literacy that can be useful in the future (Jenkins, 2006).

Despite their lack of social media skills, the parents generally reported having a positive attitude about adolescents’ social media use. From what Indonesian parents notice in their adolescent children’s daily lives, social media benefits adolescents’ social and psychological development. For example, parents report that social media allow adolescents to maintain and expand their social relationships and simultaneously learn about social roles by playing online games. Moreover, adolescents can develop their autonomy by searching for information independently. Based on their positive attitude toward social media, many parents permit their adolescents to engage in social media. This is important because parents’ positive attitudes about social media has been found to predict children’s exposure to more media use (Cingel and Krcmar, 2013), and parents’ own experiences with social media shape whether and how they monitor social media use by their adolescent children (Wallace, 2022). However, due to the limited social media skills of parents, social media may pose risks for adolescents because adolescents may lack sufficient support from knowledgeable adults (Livingstone and Byrne, 2018).

### 4.3. What are the benefits and risks of social media use for adolescents according to Indonesian adolescents and parents?

Adolescents recognize that social media can provide benefits but also contain risks. For Indonesian adolescents, social media can bring emotional benefits by enhancing their mood through pleasurable activities such as playing online games, watching YouTube, or using TikTok and social activities such as communicating, maintaining relationships, or rekindling with various people. Besides direct emotional benefits, the convenience social media provides to contact and be contacted by others allows them to maintain and establish social connections (social benefit). Exchanging news or gossip about friends or family members on social media can strengthen adolescents’ existing relationships, and having many online friends can broaden their social network. Social media also offers many Indonesian adolescents practical benefits that helps them developing hobbies, skills, but also the knowledge that is relevant to their education.

The many benefits Indonesian adolescents report gaining from social media align with those experienced by adolescents in the global North. Nine in ten social media users aged 12–15 in the United Kingdom state that social media has made them happy or helped them feel closer to their friends (OfCom, 2019). In the US, the same result was found (Anderson and Jiang, 2018). A high percentage of American adolescents report utilizing SNS for social benefits, including feeling connected to their friends’ lives, enhancing friendship diversity, and supporting each other.

The social benefits perceived by Indonesian adolescents is compatible with social media models according to which social media use can increase social capital (Verduyn et al., 2017; Bano et al., 2019; You and Hon, 2019; Tobin et al., 2020). By easing communication with close friends and family members, social media allow adolescents to accrue bonding social capital, which refers to emotional and instrumental support and companionship (Verduyn et al., 2017). This is important, as when children age into adolescence, friendships and social networks become more important to them (Wurtele, 2017; Throuvala et al., 2019b). By having many online friends, adolescents can also develop bridging social capital by being exposed to new valuable information, opportunities, and resources to develop hobbies and skills and to expand their knowledge (Putnam, 1993; Verduyn et al., 2017).

However, Indonesian adolescents are also aware of the dangers of social media. This result is consistent with research showing that certain types of social media use may negatively affect mental and physical health (Shaw et al., 2015; Chen et al., 2016; Frison and Eggermont, 2016a; Verduyn et al., 2017). One risk that adolescents often mention is that social media can be addictive. The affordances and many benefits of social media encourage adolescents to use social media more and more. For example, the ease of contacting and being contacted fosters peer attachment and information sharing between adolescents that can enrich their relationships. Unfortunately, it requires frequent or constant online presence (Throuvala et al., 2019b). Excessive social media use in adolescence is comparable to excessive and problematic smartphone use as well as parallel use of multiple devices (Griffiths et al., 2018). Bányai et al. (2017) state that it is difficult to calculate the prevalence of problematic social media use due to the variety of measurement tools used. However, several studies showed that the prevalence rate of problematic social media use in adolescents ranges from 2.6 to 9.1% (Bányai et al., 2017; Merelle et al., 2017; Wartberg et al., 2020). In Indonesia, no studies are available that provide direct evidence on the prevalence of adolescents’ problematic social media use. However, the present results suggest that social media addiction is likely also a concern in Indonesia.

Indonesian adolescents also report that social media can disrupt their daily activities and learning process. Disruption of their daily activities is reflected by forgetting to do daily activities, delaying doing homework and school assignments, suffering from sleep disturbances, and decreasing academic achievements. The disruption of their daily activities is mainly because they are too busy using social media for recreational activities, such as watching videos on YouTube or on Instagram, and playing online games. The ease by which adolescents can use social media is not accompanied by a strong ability to manage their social media usage time. Interestingly, many Indonesian adolescents do not realize this lack of time management, but their parents are well aware of this problem. A previous study has reported the same findings where most adolescents aged 12–15 in the global North consider having a good balance between screen time and doing other things (OfCom, 2019). Adolescence is a vulnerable period (Kuss and Griffiths, 2011) and adolescents’ self-regulatory processes and emotion regulation capacities are still developing (Berthelsen et al., 2017) while their social media use increases (Rideout et al., 2010). Parents and adolescents should be aware that excessive (addictive) recreational screen time may turn into maladaptive outcomes such as obesity, co-occurring psychosocial problems, and decreased wellbeing (Griffiths et al., 2018).

One interesting finding is that a small number of adolescents reported that using social media reduces their face-to-face communication. This concern is related to research on the social stimulation vs. social displacement hypothesis, according to which social media use augments (Kraut et al., 2002; Valkenburg and Peter, 2007) or displaces face-to-face interactions (Kushlev et al., 2019; Sbarra et al., 2019; Verduyn et al., 2021), respectively. It may be that social displacement effects only hold for certain individuals (Valkenburg et al., 2006), which may reflect why only a subset of adolescents expressed this concern.

Some adolescents also reported that they prefer to use social media instead of spending time with their families. Parents complained that when their children use social media, they become less sensitive to their surroundings, which is consistent with the findings from Alter (2017) and Przybylski and Weinstein (2017). From a developmental perspective, it is known that adolescents begin to develop independence from their parents and look more often to peers for support, companionship, and validation (Larson and Richards, 1991). Attachment with parents shifts to friendship and romantic relationships (Oudekerk et al., 2015). While some parents may suffer when noticing that they gradually play a less central role in their children’s life, social media may help adolescents to develop key skills they will also need later in life. By spending time with friends offline but also on social media, adolescents can develop critical interpersonal skills including building trust, empathy, and commitment (Buhrmester, 1987; Collins and Sroufe, 1999).

In this study, only a small number of adolescents (all cyber victims) mentioned cyberbullying as a risk of social media use and none of the parents mentioned it. This is surprising because the prevalence of cyberbullying is increasing (Patchin and Hinduja, 2019; Smahel et al., 2020). Cyberbullying can occur at various age ranges, but research has shown that adolescence is the period when the prevalence of cyberbullying reaches its peak (Sumter et al., 2012; Kowalski et al., 2019). According to a report from United Nations International Children’s Emergency Fund [UNICEF] (2020a), 45% of Indonesian adolescents aged 14–24 years old reported they had experienced cyberbullying. Asriani et al. (2021) confirmed that 38.41% of Indonesian adolescents admitted to be cyber offenders, and 45.35% were cyber victims. A possible explanation for the discrepancy between our qualitative findings and prior quantitative reports might be that adolescents and parents are not aware that certain acts are instances of cyberbullying if these instances are not specified. Many of them may not know that acts such as harassing or degrading other people through social media content is part of cyberbullying behavior (Rahayu, 2012; Asriani et al., 2021). Alternatively, adolescents may not consider cyberbullying to be troublesome because it is sometimes used in a playful manner for creating friendships (Samoh et al., 2019). Moreover, because it does not cause a physical impact, cyberbullying may not be taken seriously by adolescents and their parents (Hinduja and Patchin, 2021). Finally, when adolescents become victims of cyberbullying, they often do not tell their parents but instead share their experiences to their peers or keep it secret (Rahayu, 2012). This may explain why none of the parents mentioned cyberbullying as a danger of social media use.

The present study offers an in-depth understanding of how Indonesian adolescents and parents use and perceive social media. The findings are overall quite comparable to the results of studies conducted in countries in the global North, but some interesting differences were also observed. Specifically, several Indonesian adolescents reported using social media to generate income by selling items online. This way of using social media may be less prevalent in countries in the global North as this way of engaging with social media was not spontaneously mentioned by adolescents in qualitative studies conducted in the global North. Moreover, Indonesian adolescents believe that social media especially allows them to foster their social relations and only to a lesser extent to engage in self-expression. In contrast, in research conducted in the global North where adolescents are generally more individualistic, adolescents use social media often for self-promotion and documentation (Sheldon et al., 2017). Finally, while cyberbullying is perceived a major risk of social media use by parents of adolescents in the global North (Ofcom, 2021), cyberbullying was surprisingly not spontaneously mentioned as a risk factor in the present sample of Indonesian parents.

## 5. Conclusion

Indonesian adolescents and parents recognize the importance of social media in their lives. They mostly use social media for social, practical, and pleasure activities. Most adolescents report feeling skilled in utilizing social media but most parents mention that their adolescent children lack the necessary self-control to manage the amount of time they spend on social media. Most parents also mention having a low opinion about their own social media skills. Adolescents and parents agree that social media offers emotional, social, and practical benefits for adolescents. However, they also agree that social media use involves risks for adolescents, including social, emotional, and informational risks as well as displacement of more meaningful activities.

Future research is needed to extend the present findings on social media use in an Indonesian context and address the limitations of the present study. First, part of the data was collected during the outbreak of COVID-19, which may have impacted the study results. Second, the method used to recruit parent participants in this study may have led to a disproportionate representation of parents who are interested in their adolescent children’s social media use and who have a similar socioeconomic background. Third, most parents were women, and future studies are needed to better understand fathers’ use and perception of social media in Indonesia. Finally, future research is necessary to examine social media use in Indonesia outside of Bandung. This would also increase our understanding of social media use in rural areas in Indonesia.

## Data availability statement

The raw data supporting the conclusions of this article will be made available by the authors, without undue reservation.

## Ethics statement

The studies involving human participants were reviewed and approved by the Universitas Padjadjaran. Written informed consent to participate in this study was provided by the participants’ legal guardian/next of kin.

## Author contributions

EP: conceptualization, design, investigating, analyzing data, writing an original draft, writing review, and editing. KM: analyzing data, writing reviews, and editing. ZH and HA: conceptualization, design, writing review, and editing. RR: conceptualization, writing review, and editing. PV: conceptualization, analyzing data, writing an original draft, writing review, and editing. All authors contributed to the article and approved the submitted version.

## Footnotes

1 In the present paper we focus on how Indonesian adolescents and parents use and think about social media. Result on the nature of parental mediation strategies based on the same study are reported elsewhere.
